# Rapid growth of mineral deposits at artificial seafloor hydrothermal vents

**DOI:** 10.1038/srep22163

**Published:** 2016-02-25

**Authors:** Tatsuo Nozaki, Jun-Ichiro Ishibashi, Kazuhiko Shimada, Toshiro Nagase, Yutaro Takaya, Yasuhiro Kato, Shinsuke Kawagucci, Tomoo Watsuji, Takazo Shibuya, Ryoichi Yamada, Tomokazu Saruhashi, Masanori Kyo, Ken Takai

**Affiliations:** 1 Research and Development (R&D) Center for Submarine Resources, Japan Agency for Marine-Earth Science and Technology (JAMSTEC), 2-15 Natsushima-cho, Yokosuka, Kanagawa 237-0061, Japan; 2 Frontier Research Center for Energy and Resources (FRCER), School of Engineering, The University of Tokyo, 7-3-1 Hongo, Bunkyo-ku, Tokyo 113-8656, Japan; 3 Department of Earth and Planetary Sciences, School of Science, Kyushu University, 744 Motooka, Nishi-ku, Fukuoka 819-0395, Japan; 4 The Center for Academic Resources and Archives, The Tohoku University Museum, Tohoku University, 6-3 Aoba, Aramaki, Aoba-ku, Sendai, Miyagi 980-8578, Japan; 5 Department of Resources and Environmental Engineering, School of Creative Science and Engineering, Waseda University, 3-4-1 Okubo, Shinjuku-ku, Tokyo 169-8555, Japan; 6 Department of Systems Innovation, School of Engineering, The University of Tokyo, 7-3-1 Hongo, Bunkyo-ku, Tokyo 113-8656, Japan; 7 Department of Subsurface Geobiological Analysis and Research (D-SUAGR), Japan Agency for Marine-Earth Science and Technology (JAMSTEC), 2-15 Natsushima-cho, Yokosuka, Kanagawa 237-0061, Japan; 8 Laboratory of Ocean-Earth Life Evolution Research (OELE), Japan Agency for Marine-Earth Science and Technology (JAMSTEC), 2-15 Natsushima-cho, Yokosuka, Kanagawa 237-0061, Japan; 9 Department of Earth Science, School of Science, Tohoku University, 6-3 Aoba, Aramaki, Aoba-ku, Sendai, Miyagi 980-8578, Japan; 10 Center for Deep Earth Exploration (CDEX), Japan Agency for Marine-Earth Science and Technology (JAMSTEC), 3173-25 Showa-machi, Kanazawa-ku, Yokohama, Kanagawa 236-0001, Japan

## Abstract

Seafloor massive sulphide deposits are potential resources for base and precious metals (Cu-Pb-Zn ± Ag ± Au), but difficulties in estimating precise reserves and assessing environmental impacts hinder exploration and commercial mining. Here, we report petrological and geochemical properties of sulphide chimneys less than 2 years old that formed where scientific boreholes vented hydrothermal fluids in the Iheya-North field, Okinawa Trough, in East China Sea. One of these infant chimneys, dominated by Cu-Pb-Zn-rich sulphide minerals, grew a height of 15 m within 25 months. Portions of infant chimneys are dominated by sulphate minerals. Some infant chimneys are sulphide-rich similar to high-grade Cu-Pb-Zn bodies on land, albeit with relatively low As and Sb concentrations. The high growth rate reaching the 15 m height within 25 months is attributed to the large hydrothermal vent more than 50 cm in diameter created by the borehole, which induced slow mixing with the ambient seawater and enhanced efficiency of sulphide deposition. These observations suggest the possibility of cultivating seafloor sulphide deposits and even controlling their growth and grades through manipulations of how to mix and quench hydrothermal fluids with the ambient seawater.

Ancient volcanogenic massive sulphide (VMS) deposits are exploited on land as one of the major Cu-Pb-Zn(±Au ± Ag) resources. These syngenetic, stratiform hydrothermal deposits occur worldwide and have formed throughout geologic time^1^,^2^,^3^. Kuroko-type sulphide deposits are VMS deposits interpreted as the ancient counterpart of seafloor hydrothermal deposits that are being formed today in a back-arc setting^4^,^5^,^6^. Active hydrothermal deposits of this type were investigated in 2000 during Ocean Drilling Program (ODP) Leg 193 in the Manus Basin^7^ and in 2010 during Integrated Ocean Drilling Program (IODP) Expedition 331, in the Iheya-North field in the Okinawa Trough^8^,^9^ (Fig. 1).

IODP Expedition 331 obtained samples of polymetallic massive sulphides and inadvertently created four artificial hydrothermal vents in boreholes at drilling sites C0013, C0014 and C0016^8^,^9^ (Fig. 1). Subsequent visits by research cruises have collected samples of hydrothermal fluids and mineral deposits from these very young, artificially produced chimneys no more than two years old (hereafter called infant chimneys) using a remotely operated vehicle (ROV)^10^. At Hole C0016A on the top of the North Big Chimney (NBC) mound, which was discharging high-temperature hydrothermal fluid (up to 310 °C^10^,^11^,^12^) at the time of IODP Expedition 331, rapid chimney growth has been documented at the drilling site (Figs. 2a–c). Although the upper part of this growing chimney was broken twice during the collection of hydrothermal fluids (Fig. 2b), it grew to a height of 15 m within 25 months^10^.

In this study, we examined solid samples of 10 fragments from seven infant chimneys and two samples of hydrothermal fluid collected in 2011 and 2012 by ROV *Hyper Dolphin* during cruises KY11-02, NT11-15, NT11-16, NT12-06 and NT12-27 of R/Vs *Kaiyo* and *Natsushima* (Tables S1 and S2). Four solid samples were taken from chimneys 20–30 cm high that were growing from the wellhead corrosion caps installed on the guide bases of Holes C0016B and C0013E (samples HPD1247R01, HPD1312G03, HPD1317R01 and HPD1449R01) (Fig. S1 and Table S1). One chimney (sample HPD1449R01-pipe) formed within the stainless steel pipe of the corrosion cap on Hole C0016B, and another (sample HPD1450R01) formed on the sediment-covered seafloor several metres from Hole C0014G. Four chimney fragments (HPD1355G01–G04) were obtained from a large infant chimney at Hole C0016A on the NBC mound (Fig. S2), and two hydrothermal fluid samples were taken from the same vent. Post-drilling observations of chimney growth have been published along with ROV dive photos^10^.

## Results

Two infant chimneys (samples HPD1312G03 and HPD1450R01) were dominated by Ca-sulphate minerals such as anhydrite and gypsum. These two sulphate-dominant chimneys were very fragile and their original spire structures were destroyed during the collection of samples. Only two samples, HPD1247R01 and HPD1317R01, were recovered with the original chimney structures. The basal side of sample HPD1247R01 is about 6 cm in diameter, and it encompasses a hollow tube (2 cm wide), partly filled with white sulphate minerals, from the centre of the chimney. The area surrounding this hollow tube is dominated by sulphide minerals, and this sulphide-rich region is surrounded by the outermost thin brown sulphate-rich rind (1 mm thick). Sample HPD1317R01 is from a sulphide-rich chimney with a thin (1 mm) brown sulphate-rich rind (Fig. S3). At least five hollow tubes, with widths ranging from 1 to 3 cm, occur within the sulphide-rich part, and chalcopyrite is concentrated around these hollow tubes (Fig. S3). The other samples (from dives HPD1355 and HPD1449) include fragments of the chimney because the chimney on Hole C0016A was too large to wholly collect safely by ROV or original structures were destroyed during the sampling operations. We report below petrographic features such as constituent minerals and mineral textures of both the sulphate- and sulphide-rich parts of chimneys. Observations during dive HPD1355 (Fig. S2) suggest that the top and outermost parts of the sulphide-rich chimney were composed of Ca-sulphate-rich rinds.

### Petrographic features of sulphate-rich material

The sulphate-rich samples consist of euhedral anhydrite and euhedral to subhedral gypsum with less than 5 vol% total of euhedral to subhedral sphalerite, galena, chalcopyrite, anglesite (PbSO_4_), Zn-sulphate mineral and talc. Some portions of the sulphate minerals, up to 1.5 mm size, are replaced or overgrown by sulphide minerals such as sphalerite, galena and chalcopyrite with grain sizes ranging from several to 50 μm (Figs. S4a–S4e). More than half of the sphalerite and galena replacing the rims of Ca-sulphate minerals exhibit dendritic texture (Figs. S4e), and the sphalerite in this setting is closely associated with acicular anglesite crystals up to 40 μm long (Fig. S4f and S4g). Radially oriented talc and amorphous silica were observed surrounding sulphide minerals and as overgrowths on Ca-sulphates (Figs. S4h and S4i). Sulphide minerals and talc also occur where Ca-sulphate minerals dissolved (Figs. S4j and S4k). Acicular or tabular unidentified Zn-sulphate minerals (<300 μm) displaying H and Cl peaks in energy dispersive X-ray spectrometer (EDS) analyses were closely associated with Ca-sulphate minerals (Figs. S4k and S4l).

Because the solubility of anhydrite varies inversely with temperature^13^, dissolution and replacement of Ca-sulphates is related to a growth mechanism of chimneys, as commonly observed in seafloor hydrothermal deposits^14^,^15^,^16^. Chimneys initially form as Ca-sulphate-rich mineral walls. Within the shelter of these sulphate walls, the temperature of hydrothermal fluids can rise enough to permit precipitation of sulphide minerals^14^, as the sulphate minerals of the outer wall undergo further dissolution and replacement.

### Petrographic features of sulphide-rich material

**Figure 2:**
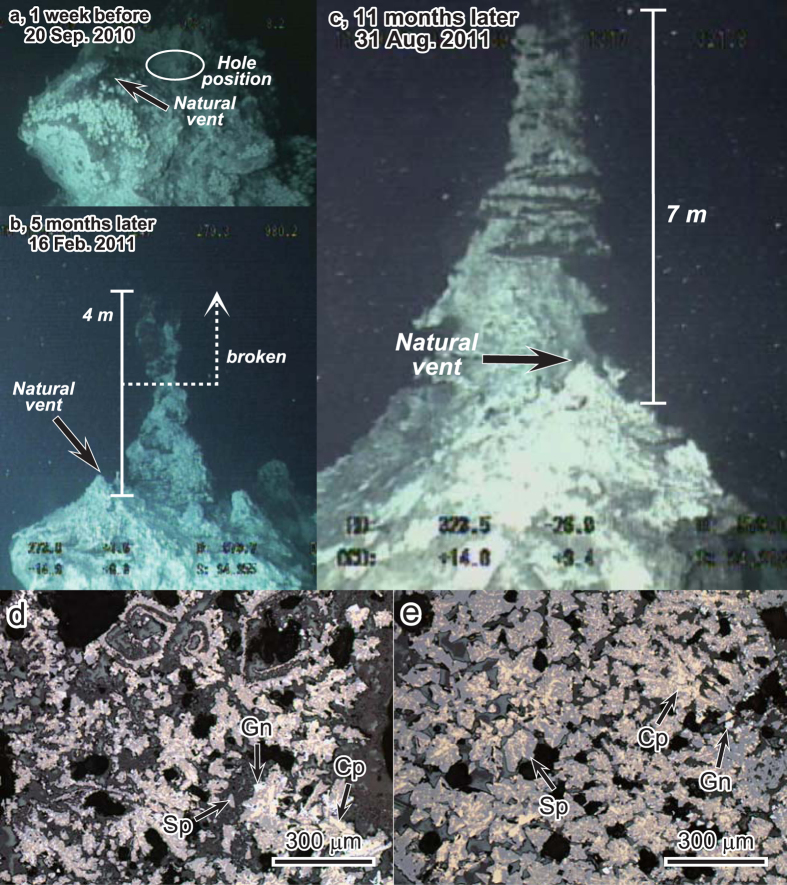
Camera images and photomicrographs of the infant chimney. Camera images of the infant chimney on Hole C0016A (NBC mound) in the Iheya-North field, Okinawa Trough, captured (**a**) 1 week before, (**b**) 5 months after and (**c**) 11 months after drilling operations during IODP Expedition 331. These camera images were taken by S. Kawagucci, T. Watsuji, J.-I. Ishibashi and K. Takai. (**d,e**) Reflected-light photomicrographs of samples collected from the infant chimney at Hole C0016A during dive HPD1355. Rims of Ca-sulphates are replaced by the sulphide minerals chalcopyrite (Cp), sphalerite (Sp) and galena (Gn) in the outer part of a flange sample (**d**, HPD1355G02). The inner part of a flange sample (**e**, HPD1355G03) shows a dendritic texture of chalcopyrite, sphalerite and galena.

**Figure 3:**
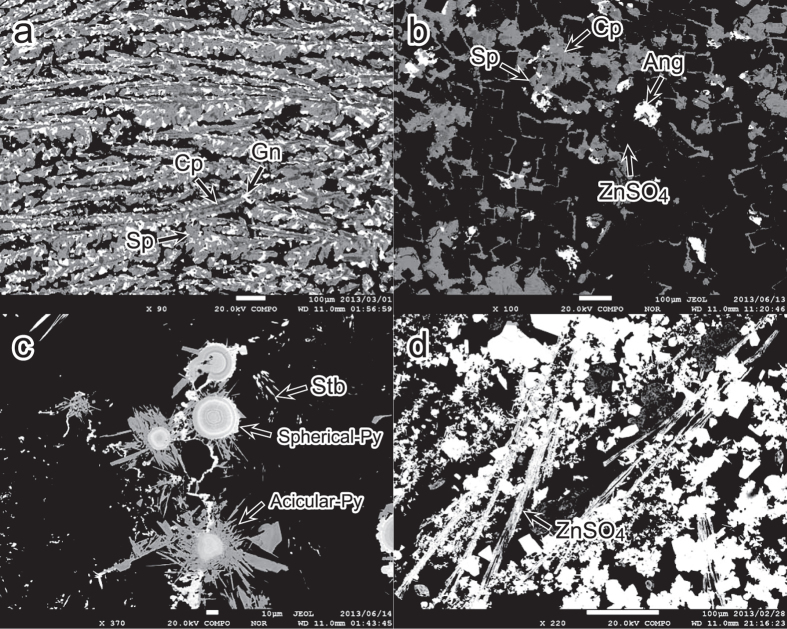
Back-scattered electron images of infant chimney samples. (**a**) Dendritic textures of chalcopyrite, sphalerite and galena (sample HPD1317R01). (**b**) Encrustation and pseudomorph texture of sulphide minerals after sulphate minerals (sample HPD1247R01). (**c**) Spherical pyrite crystals of alternating pyrite and Pb-As-Ag-Sb-Cu(±Mn ± Zn)-rich layers, restricted to the outermost parts of the sulphide-rich chimney (sample HPD1449R01). (**d**) Acicular Zn-sulphate minerals in the outer part of the infant chimney (sample HPD1317R01). Mineral abbreviations: Ang = anglesite, Cp = chalcopyrite, Gn = galena, Py = pyrite, Sp = sphalerite, Stb = stibnite.

More than 95 vol% of the constituent minerals in the sulphide-rich parts of the chimneys were euhedral to subhedral chalcopyrite, sphalerite and galena with a dendritic texture. X-ray diffraction patterns suggest that some fractions of the Zn sulphide minerals consist of wurtzite, a polymorph of sphalerite, as a minor phase and wurtzite was detected from all sulphide-rich samples. The grain size of these minerals increases towards the interior of the chimney structure from typically 10 μm in the outermost part to 100 μm in the central part. In the outer parts, the dendritic texture consisting only of sphalerite and galena were also observed. The area of dendritic texture includes 10–20 vol% porosity without considering the numerous hollow hydrothermal paths (about 100 μm wide), as determined by microscopic observations, but some portions of the pore spaces are filled with anglesite and fine-grained (several to 50 μm) euhedral anhydrite, acicular barite, talc and amorphous silica that together total less than 5 vol% of the chimney volume (Figs. 2d,e and 3a, S5c–e and S6). The outer parts of these dendritic zones contain voids left by dissolution of sulphate minerals, filled by sulphide pseudomorphs (Figs. 2d and 3b, S5a, S5i and S5j) as well as acicular or anhedral anglesite, acicular or tabular Zn-sulphates and amorphous silica (Figs. 3b, S5a, S5i and S5j).

Spherical and acicular pyrite crystals with grain sizes ranging from several to 200 μm occur exclusively in the outer parts of the chimneys (Figs. 3c, S5b and S5k), and the spherical crystals contain alternating layers several micrometres thick of pyrite and material enriched in Pb-As-Ag-Sb-Cu(±Mn ± Zn) with up to 7 wt% Ag. Acicular pyrite crystals (about 20 μm in length) are slightly arsenical with up to 3.1 wt% As. These pyrite crystals are closely associated with amorphous silica and rarely observed stibnite that contains Mn (~3.1 wt%) and Ag (~1.2 wt%) (Figs. 3c, S5g and S5h). As spherical pyrite crystals are considered to form under conditions of sulphur supersaturation^17^, we infer that they precipitated as the hydrothermal fluid cooled and sulphur saturation increased. From the inner to the outer side of the chimney, sulphide-rich sample HPD1449R01 exhibits four mineralogical zones: (1) coarse-grained (typically 100 μm), dendritic chalcopyrite and sphalerite with galena, (2) fine-grained (typically 10 μm), dendritic chalcopyrite and sphalerite with galena, as well as the dendritic textures consisting only of sphalerite and galena, (3) amorphous silica with peaks for alkali and alkali earth elements (Na, Mg, K and Ca) and Al in EDS analyses, and (4) abundant barite along with spherical and acicular pyrite (Figs. S5f and S5g). Acicular or anhedral anglesite and acicular or tabular Zn-sulphate occur in the outermost parts of the sulphide-rich chimney as well as in sulphate-rich parts (Figs. 3d and S5j).

### Electron microscopic observations

Scanning electron microscope (SEM) and transmission electron microscope (TEM) observations show that highly crystalline sphalerite is characterised by a paucity of stacking faults and by tetrahedral crystals with well-developed {111} faces (Figs. S7a and S8a). In contrast, small sphalerite crystals occurring paragenetically with wurtzite have platy or tabular forms with (111) twin defects (Figs. S7b and S8b). Wurtzite occurs in hexagonal columns that are easily distinguished from sphalerite (Figs. S7c and S8c). Some dendritic sphalerite crystals exhibit intergrowth (or co-precipitate) texture with chalcopyrite and galena (Figs. S5l and S7d–f). Sphalerite crystals have a tetrahedral form with well-formed {111} faces (Fig. S7e) repeated. Cut surfaces of these sphalerite crystals display a co-precipitate texture with chalcopyrite and galena (Figs. S5l and S7f). Back-scattered electron images and TEM observations reveal that sphalerite and chalcopyrite crystals are in an epitaxial relationship in which the *a*-axis of sphalerite is parallel to the *a*- and *c*-axes of chalcopyrite.

### Whole-rock geochemistry

**Figure 4:**
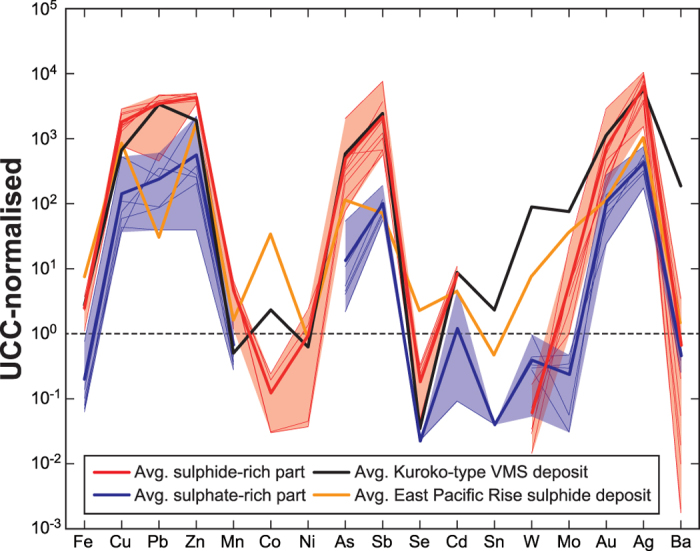
Composition of infant chimney. Elemental concentrations, relative to upper continental crust (UCC)^49^, of sulphide-rich and sulphate-rich infant chimneys together with an average Kuroko-type VMS deposit on land^50^ and East Pacific Rise sulphide deposit^51^.

The samples from the sulphide-rich parts of infant chimneys (Table S1) are enriched in metals compared with sulphate-rich parts, with average concentrations of 4.5 wt% Cu, 6.9 wt% Pb, 30.3 wt% Zn and 8.7 wt% Fe as well as several hundred ppm of Ag, Cd, Sb, Ba and Bi, and 1.35 ppm Au (Fig. 4 and Table S1). Ratios of major elements (Cu, Zn and Pb) likely reflect the composition of the host hydrothermal fluid (Fig. 4 and Tables S1, S2), but Mn, alkali and alkali earth elements (Na, K and Ca) that are incompatible with sulphide minerals tend to be released into seawater (Fig. 4 and Tables S1, S2). The inner side of the hollow tube (except for the sulphate-rich samples HPD1312G03, HPD1355G01 and HPD1450R01), corresponding to the hydrothermal fluid path, is commonly dominated by chalcopyrite. Microscopic observations reveal that chalcopyrite, sphalerite and wurtzite precipitated in an earlier stage, and hence at a higher temperature, than galena^18^,^19^,^20^. Because the sulphide-rich infant chimneys at Hole C0016 were formed by high-temperature hydrothermal fluids up to ~310 °C^10^, they have slightly higher Cu and Zn concentrations than average Kuroko-type VMS deposits on land, along with lower Co, As, W and Mo concentrations (Fig. 4).

## Discussion

The large infant chimney on the NBC mound (Hole C0016A) is dominated by dendritic chalcopyrite, sphalerite (±wurtzite) and galena (Figs. 2e and 3a) except for its top and outermost parts (Fig. S2). It contains less pyrite and silicate minerals than the subseafloor sulphide sample collected from the flank of the NBC mound during IODP Expedition 331 (Sample 331-C0016B-1L-1, 18–20 cm)^8^,^21^,^22^. This difference is consistent with the stratigraphic sequence of Kuroko-type VMS deposits, which typically consists of a basal zone of Keiko (siliceous ore in the stockwork zone corresponding to mineralised/altered volcanic rock), succeeded in ascending order by Oko (yellow ore), Kuroko (black ore), barite ore and haematite-quartz-rich sedimentary rock^4^,^5^,^6^. Keiko, Oko and Kuroko ores mainly consist of quartz + pyrite + chalcopyrite, pyrite + chalcopyrite and sphalerite + galena + chalcopyrite, respectively, and the proportion of pyrite usually decreases in ascending order^4^,^5^,^6^. Thus, the Cu-Pb-Zn-rich infant chimneys are consistent with the uppermost part of the sulphide zonal structure in a Kuroko-type VMS deposit. Indeed, chimney-like structures are often reported^23^ from the upper parts of Kuroko-type VMS deposits in the Hokuroku area, Japan, which is the type locality of Kuroko deposits.

The relatively low average As concentrations of 409 ppm in the sulphide-rich parts of the infant chimneys except for one high-As sample (3050 ppm, HPD1355G04-H) are consistent with the facts that As-bearing minerals such as realgar and orpiment precipitate later during chimney formation and As is enriched in lower temperature parts like as commonly observed at hydrothermal deposits in a back-arc setting^18^. Aside from this small difference, sulphide-rich infant chimneys are geochemically similar to average Kuroko-type VMS deposits on land and distinct from average sulphide deposits along the East Pacific Rise (Fig. 4). Because pyrite and silicate minerals have no economic value, and because high As and Sb concentrations make smelting of sulphide materials more costly, the low concentrations of these components in infant chimneys as well as high average concentrations of 4.5 wt% Cu, 6.9 wt% Pb, 30.3 wt% Zn in the sulphide-rich parts mean that such chimneys have the potential to represent high-grade sulphide deposit.

The infant chimney at Hole C0016A (Fig. 2) grew to a height of 15 m in 25 months^10^ even though its upper half was broken off twice during this period. Four mineral samples (HPD1355G01–G04) obtained from this chimney were fragments of several tens of centimetres in size, and detailed structures within this chimney cannot be observed. However, on the basis of the cross-sectional image of sample HPD1317R01 and dive photos during dive HPD1355 (Figs. S2 and S3), assuming a conical shape 3 m across and 15 m high with a sulphate-rich outer layer of 5 cm thick, the sulphide-rich part of the chimney (2.5 g/cm^3^ average dry density determined from dendritic sulphide-rich sample cubes) is estimated to grow by 39.5 t/year and reach a total mass of 82.3 t. The dive photos (Fig. S2) suggest that the inner part of the 15 m high large chimney is dominated by sulphide-rich parts; thus, our estimate should be valid as a first-order approximation. This rate of chimney growth (metal accumulation rate) is almost equivalent to rates of entire vent fields containing tens or hundreds of individual chimneys, such as the Main Endeavour Field on the Juan de Fuca Ridge (50 t/year) and the Rainbow Field on the Mid-Atlantic Ridge (35 t/year)^24^. The chimney growth rate of the NBC mound represents an annual production of 1.8 t Cu, 2.7 t Pb, 12.0 t Zn and 3.4 t Fe, based on an average concentration of 4.5% Cu, 30.3% Zn, 6.9% Pb and 8.7% Fe in the sulphide-rich parts (Table S1). Growth rates of chimneys at the artificial hydrothermal vents seem to depend on the presence of long casings beneath the guide base. Hole C0016A is a bare hole without casing^8^,^9^, whereas Holes C0013E and C0014G have stainless steel casing pipes extending 40.2 and 117.6 metres below the seafloor (mbsf)^8^,^9^, respectively. These pipes strongly focus the hydrothermal fluids, and the resulting unidirectional high-velocity flows inhibit the growth of chimneys. The enlarged bare orifice of Hole C0016A, in contrast, appears to promote turbulent mixing of fluid and seawater and to lower the velocity of discharge, possibly leading to efficient precipitation of sulphide minerals^10^. Hydrothermal discharge at Hole C0016B, with a casing pipe reaching only 1.0 mbsf, has continued at least 40 months after drilling and produced sulphide-rich infant chimneys. We ascribe such long-lived hydrothermal discharge (>40 months) to shimmering of hydrothermal fluids or leakage within and around the corrosion cap or guide base from the annulus between the drilled hole wall (~50 cm in diameter) and the installed casing pipe (5.5 inch = ca. 14.0 cm in diameter).

Very rapid growth of sulphide chimneys (>1.2 m/day) has been observed at the Endeavour Segment on the Juan de Fuca Ridge in association with a swarm of microearthquakes, although the chimneys collapsed shortly after the earthquake swarm^25^. Moreover, chimneys in the Sasquatch hydrothermal field in the Endeavour Segment have been observed to grow rapidly (10 m/year) in response to a dyking event in 2005^26^. Rapid growth of chimneys was necessarily associated with seismic events in the former case or volcanic events in the case of the Sasquatch hydrothermal field, and not with artificial hydrothermal vents. Chimney growth rates and metal accumulation rates in sulphate/sulphide chimneys have been estimated using short-lived (~150 years) or longer lived (up to 10^5^ years) U-series disequilibrium dating techniques^24^,^27^,^28^,^29^. An artificial hydrothermal vent has an advantage in this respect given that the mineralisation interval corresponding to the chimney age can be easily determined from the time since the drilling operation. Moreover, the ongoing rate of chimney growth can be estimated repeatedly by trimming the chimney structure during ROV dive expeditions.

Given a flow velocity of 1 m/s for hydrothermal fluid at Hole C0016A, as documented for the natural NBC vent before drilling operations^30^, together with the 8.5 inch (ca. 21.6 cm) drilled hole diameter^8^ and the 1.0 g/cm^3^ density of the hydrothermal fluid, the flow rate of hydrothermal fluid is estimated to be 36.6 kg/s. Given the metal concentrations in the unfiltered hydrothermal fluid of 0.54 ppm Cu, 1.61 ppm Pb, 5.07 ppm Zn and 4.07 ppm Fe (Table S2), the corresponding metal discharge rates are estimated to be 0.62 t/year of Cu, 1.9 t/year of Pb, 5.9 t/year of Zn and 4.7 t/year of Fe. Assuming a 5% depositional efficiency^24^ from the hydrothermal fluid, the metal accumulation rates would be 5% of the discharge rates; that is, 0.031 t/year of Cu, 0.093 t/year of Pb, 0.29 t/year of Zn and 0.23 t/year of Fe, which are one or two orders of magnitude lower than those we estimated from the growth rates and metal concentrations of infant chimneys (1.8 t Cu, 2.7 t Pb, 12.0 t Zn and 3.4 t Fe per year) (Table S1). Even assuming a 100% depositional efficiency, discrepancies still remain. These suggest that we might overestimate the metal accumulation rate on the basis of the chimney growth rate, because there should be numerous hollow hydrothermal paths as well as some sulphate-rich parts within the chimney structure, which would decrease the estimated metal accumulation rate. Thus, the metal accumulation rate estimated for a circular cone structure with a sulphate-rich outer layer 5 cm thick is considered to be a maximum estimate, although the infant chimney on Hole C0016A was broken twice and this infant chimney would be higher than 15 m within 25 months. Another probable explanation is that the discrepancies in metal accumulation rates can be attributed to underestimation of the flow velocity or the drilled hole diameter, because the 1 m/s flow velocity for hydrothermal fluid was based on visual observations before drilling, and we have no precise data on the flow velocity of the hydrothermal discharge just after the drilling. Moreover, Hole C0016A is a bare hole without casing, and its diameter would necessarily exceed the 21.6 cm (8.5 inch) diameter of the rotary core barrel^8^; indeed, the actual hole diameter was greater than 50 cm^10^. Calculations based on a 50 cm hole diameter instead of the 21.6 cm (8.5 inch) exact diameter of a rotary core barrel yields a 5.4 times hydrothermal flow rate, which reduces the discrepancy of estimations. Because high-velocity flows disperse sulphate/sulphide minerals and inhibit the growth of chimneys, as observed at Hole C0014G^10^, the rapid chimney growth at Hole C0016A might be an effect of the great size of the bare hole (>50 cm) compared to that of the natural hydrothermal conduits. Because the rapidly grown chimney at Hole C0016A has many flange structures^10^ that could trap hydrothermal fluids and enhance precipitation of sulphides, it is also likely that the relatively slow flow velocity (less than 1 m/s) from the unnaturally large bare hole meant that metals effectively accumulated from the hydrothermal fluids. The Pb accumulation rates estimated from the chimney growth rate and hydrothermal flux would be identical at a flow velocity of 0.27 m/s, a hole diameter of 50 cm and 100% depositional efficiency. Assuming that half of the sulphide-rich part of the Hole C0016A chimney was hollow, as indicated in the cross-sectional image of sample HPD1317R01 (Fig. S3), the Pb accumulation rate estimated from the chimney growth rate and that estimated from the hydrothermal flux would be identical at a flow velocity of 0.14 m/s and 100% depositional efficiency. Since hydrothermal fluid flux is primarily controlled by heat source, the large artificially produced bare hole more than 50 cm in diameter should decrease the flow velocity. Thus, the combination of an unnaturally large hydrothermal conduit (a drilled hole larger than 50 cm) and a slow flow velocity, producing slow mixing with ambient cold seawater, may be the key factor producing such an anomalously high chimney growth rate.

Although there are uncertainties in the flow velocity of the hydrothermal fluid and the hole diameter, rapid hydrothermal discharge associated with artificial vents can transport large amounts of valuable metals into the seawater. This fact inspires the possibility of “cultivating” sulphide deposits on artificial hydrothermal vents by deploying suitable porous media on the top of the casing pipes.

We propose that Kuroko-type deposits could be cultivated and harvested by using apparatus installed on artificial hydrothermal vents^31^ for effectively recovery of mineral resources from hydrothermal fluids. Regulating hydrothermal fluids with valves may enable control of not only the flow rate and temperature of hydrothermal fluid but also the path and location of mineral deposition in the cultivation apparatus. Furthermore, artificial controls on the chemical and physical properties of hydrothermal fluid could potentially enrich deposits in selected elements. For instance, Au is dissolved in hydrothermal fluid as bisulphide or chloride complexes, depending on temperature, oxygen fugacity (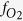) and pH conditions^32^,^33^. If the apparatus could produce the boiling and phase separation conditions observed at the hydrothermal systems in the Okinawa Trough^34^,^35^,^36^, where raising the pH and decreasing the H_2_S concentration of the hydrothermal fluid by degassing CO_2_ and H_2_S would promote precipitation of Au derived from both bisulphide and chloride complexes^32^.

For another example, we consider a possible recovery scheme for Zn. As crystals grow, different surfaces with different Miller-index numbers grow at differing rates, and control of this phenomenon is used in producing synthetic quartz crystals by favouring the bottom (0001) crystal face over the slower-growing rhombohedral (10
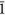
1) or prismatic (10
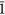
0) surfaces^37^,^38^,^39^. Sphalerite crystals in the infant chimneys typically have a tetrahedral shape enclosed by four (111) surfaces (Figs. S7a and S8a). These faces have small growth rates that are difficult to increase. However, infant chimneys also contain the metastable Zn sulphide mineral wurtzite, which consists of basal (000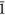) and prismatic (10
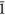
0) planes (Figs. S7b,c and S8c). Although the (0001) plane of wurtzite is a singular face like the (111) plane in sphalerite, wurtzite in the infant chimney favours the (000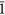) surface, allowing it to grow more rapidly than sphalerite. Thus, maintaining conditions favourable for wurtzite such as low sulphur fugacity, reducing and high supersaturation degree of ZnS in the hydrothermal fluid^40^,^41^ may enable more effective and rapid precipitation of Zn.

Another possible application that would favour Cu extraction relies on manipulating crystal nucleation, which needs a high activation free energy. In the absence of nucleation, valuable metals in hydrothermal fluid may be lost to seawater. The barrier of high activation free energy is often overcome by the use of seed crystals, as commonly used in the manufacture of quartz crystals^37^,^38^,^39^. Sphalerite and chalcopyrite have isomorphous crystal structure and are well known for displaying epitaxial growth relationships in which they crystallize simultaneously^42^. Microscopic observations (Figs. S5l and S7f) indicate that simultaneous precipitation of sphalerite and chalcopyrite crystals is controlled by epitaxial growth on a parent phase of sphalerite. This suggests that chalcopyrite crystals can be effectively precipitated and recovered by using sphalerite seed crystals. Similar epitaxial relationships exist between wurtzite and chalcopyrite, and between galena and pyrite; thus, the use of seed crystals within the cultivation apparatus may allow enrichment in several selected elements. These speculative methods for selected metal element precipitation would need to be addressed by further experimental studies aimed at quantifying the enhanced metal accumulation rates.

Compared to conventional mining of naturally formed chimney and mound sulphide deposits, a sulphide harvesting system based on artificial hydrothermal vents would offer the possibility of greatly decreased environmental impacts on the surrounding deep-sea ecosystems because the cultivation sites would recover quickly (<40 months) after the initial drilling operations^43^. It would also be suitable for the relatively shallow seafloor hydrothermal deposits found in back-arc settings. The Iheya-North field in the Okinawa Trough, at ~1000 m water depth, is much shallower than the typical hydrothermal sites along mid-ocean ridges (~2500–3000 m^44^). Exploration costs should be negligible because artificial hydrothermal vents can be produced at known hydrothermal sites. Moreover, artificial hydrothermal vents promise to be semi-permanent, remaining productive until the hydrothermal fluid reservoir (heat source) is depleted or the fluid path is plugged by scales, which may be periodically removed as they are at geothermal power plants (although remotely operated methods of scale removal would need to be developed). Although there are not yet any data from the Okinawa Trough on the duration of the high-permeability condition required for rapid chimney growth, the infant chimney at Hole C0016A has continued to grow for at least 25 months^10^, which is much longer than the days-long episode of rapid chimney growth observed at the Juan de Fuca Ridge^25^. The subseafloor stratigraphy at the Iheya-North Knoll, composed of terrigenous or hemipelagic sediment and felsic, pumiceous volcaniclastics^8^ is favourable for long-lived hydrothermal activity. In conclusion, the cultivation of high-grade sulphide deposits through artificial hydrothermal vents may be compared to industrial aquaculture.

The roughly estimated growth rate of 39.5 t/year from the rapidly grown chimney at Hole C0016A is equivalent to a daily formation of ca. 0.11 t of sulphide deposit. Compared to the typical mining production rate from a VMS deposit on land of several thousand tons of ore per day, the sulphide deposit accumulation rate from one artificial hydrothermal vent is three or four orders of magnitude smaller. An artificial vent could compensate for this difference with more effective sulphide mineral formation and recovery methods, using a cultivation apparatus with a casing pipe to enhance the natural hydrothermal flux and metal accumulation rate because the large sulphide-rich chimney on Hole C0016A was formed on the bare hole without casing pipes. At this time, there are certainly scientific and technical barriers to overcome, but such a Kuroko deposit cultivating method would enable the recovery of high-grade sulphide materials with dendritic textures, abundant wurtzite and spherical pyrite crystals with chemical zoning, features found at the initial mineralisation stage that are usually erased by oxidative dissolution^45^,^46^ and are rarely preserved in the geological record.

## Methods

### ICP-QMS analysis

The composition of hydrothermal fluid and infant chimneys was measured by inductively coupled plasma quadrupole mass spectrometry (ICP-QMS, Agilent 7500c) at the University of Tokyo^47^. Approximately 0.1 g of a powdered sample from an infant chimney was digested in a solution of 0.8 mL HClO_4_, 2 mL HF and 4 mL HNO_3_ at 130 °C for 24 h. After drying this solution by stepwise heating, 6 mL aqua regia was added and then heated at 160 °C overnight. This solution was dried, then 4 mL HNO_3_, 1 mL HCl and 5 mL de-ionised Milli-Q water were added and heated at 160 °C for at least 3 h. Finally, the sample solution was appropriately diluted with de-ionised Milli-Q water and used for the ICP-QMS analysis.

### EPMA analysis

Chemical composition of constituent minerals was determined by a field emission-electron probe microanalyser (FE-EPMA; JEOL JXA-8530F) at Kyushu University. Accelerating voltage was 20 kV and beam current was 10 nA. The incident electron beam was focused to be 100 nm in diameter and counting time was 40 s for each element. The acquired X-ray intensities were corrected by the ZAF method. X-ray mapping was conducted with 20 kV accelerating voltage, 50 nA beam current, 0.6 μm scan distance and 10 ms X-ray peak acquisition time per point.

### SEM, TEM and EBSD observations

Mineral grains were characterised using an SEM (JEOL JEM-6510) at Tohoku University. The samples for TEM analysis were prepared by argon-ion milling. A TEM (JEOL JEM-2010) at Tohoku University with an accelerating voltage of 200 kV was used to observe the crystal structure of ZnS minerals. Selected-area electron diffractions, used to examine partial diffraction patterns for areas 400 nm in diameter, were obtained with the TEM. Electron back-scattered diffraction (EBSD) analyses were carried out using an SEM (Hitachi S-3400N) with HKL Technology and Channel 5 software at Tohoku University.

## Additional Information

**How to cite this article**: Nozaki, T. *et al.* Rapid growth of mineral deposits at artificial seafloor hydrothermal vents. *Sci. Rep.* **6**, 22163; doi: 10.1038/srep22163 (2016).

## Supplementary Material

Supplementary Information

## Figures and Tables

**Figure 1:**
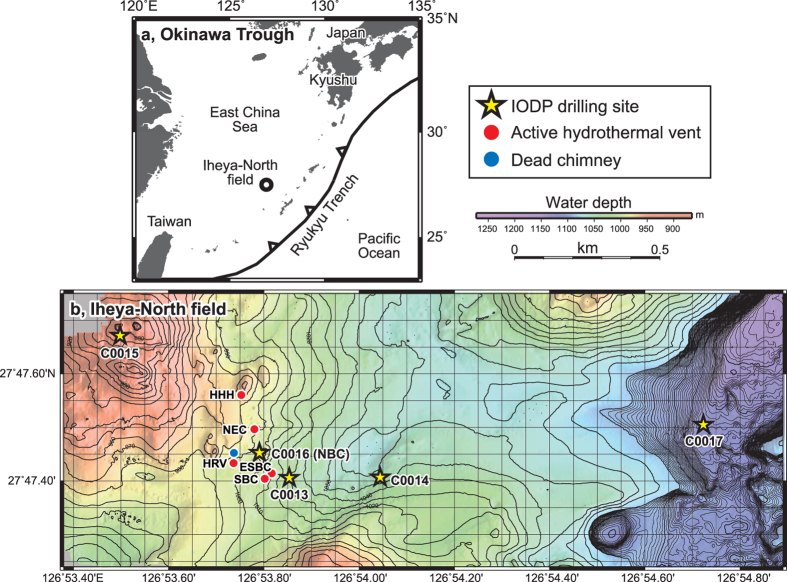
Location of the Iheya-North field, Okinawa Trough. (**a**) Regional map. Heavy line is the megathrust at the boundary between the Philippine Sea and Eurasia plates; teeth are on upthrown side. (**b**) Bathymetry of the Iheya-North field with locations of five drilling sites from IODP Expedition 331^8^,^9^ and hydrothermal chimneys^10^. Depth contours are at 10 m intervals. This bathymetric map was produced by AUV *Urashima* survey during YK07-07 cruise^10^,^48^ of R/V *Yokosuka* in May, 2007. The applied multi-narrow beam echo sounder was a SEABAT 7125 whose frequency and beam width were 400 kHz and 1.0 degree × 0.5 degree (fore-aft × athwart).
