# Supplementary material for: Rapid growth of mineral deposits at artificial seafloor hydrothermal vents
Source: Sci Rep. 2016 Feb 25;6:22163. doi: 10.1038/srep22163 (PMC4766430; doi:10.1038/srep22163)
Supplement: Supplementary Information [file srep22163-s1.pdf]

Rapid growth of mineral deposits at artificial seafloor hydrothermal vents

Tatsuo Nozaki[1,2*], Jun-Ichiro Ishibashi[3], Kazuhiko Shimada[3], Toshiro Nagase[4], Yutaro Takaya[1,5], Yasuhiro Kato[1,2,6], Shinsuke Kawagucci[1,7,8], Tomoo Watsuji[7], Takazo Shibuya[1,8], Ryoichi Yamada[9], Tomokazu Saruhashi[10], Masanori Kyo[10] and Ken Takai[1,7,8]

[1]Research and Development (R&D) Center for Submarine Resources, Japan Agency for Marine-Earth Science and Technology (JAMSTEC), 2-15 Natsushima-cho, Yokosuka, Kanagawa 237-0061, Japan.

[2]Frontier Research Center for Energy and Resources (FRCER), School of Engineering, The University of Tokyo, 7-3-1 Hongo, Bunkyo-ku, Tokyo 113-8656, Japan.

[3]Department of Earth and Planetary Sciences, School of Science, Kyushu University, 744 Motooka, Nishi-ku, Fukuoka 819-0395, Japan.

[4]The Center for Academic Resources and Archives, The Tohoku University Museum, Tohoku University, 6-3 Aoba, Aramaki, Aoba-ku, Sendai, Miyagi 980-8578, Japan.

[5]Department of Resources and Environmental Engineering, School of Creative Science and Engineering, Waseda University, 3-4-1 Okubo, Shinjuku-ku, Tokyo 169-8555, Japan.

[6]Department of Systems Innovation, School of Engineering, The University of Tokyo, 7-3-1 Hongo, Bunkyo-ku, Tokyo 113-8656, Japan.

[7]Department of Subsurface Geobiological Analysis and Research (D-SUAGR), Japan Agency for Marine-Earth Science and Technology (JAMSTEC), 2-15 Natsushima-cho, Yokosuka, Kanagawa 237-0061, Japan.

[8]Laboratory of Ocean-Earth Life Evolution Research (OELE), Japan Agency for Marine-Earth Science and Technology (JAMSTEC), 2-15 Natsushima-cho, Yokosuka, Kanagawa 237-0061, Japan.

[9]Department of Earth Science, School of Science, Tohoku University, 6-3 Aoba, Aramaki, Aoba-ku, Sendai, Miyagi 980-8578, Japan.

[10]Center for Deep Earth Exploration (CDEX), Japan Agency for Marine-Earth Science and Technology (JAMSTEC), 3173-25 Showa-machi, Kanazawa-ku, Yokohama, Kanagawa 236-0001, Japan.

***Correspondence:** Correspondence and requests for materials should be addressed to T. N. (email: nozaki@jamstec.go.jp).

Supplementary Information

Supplementary Figures

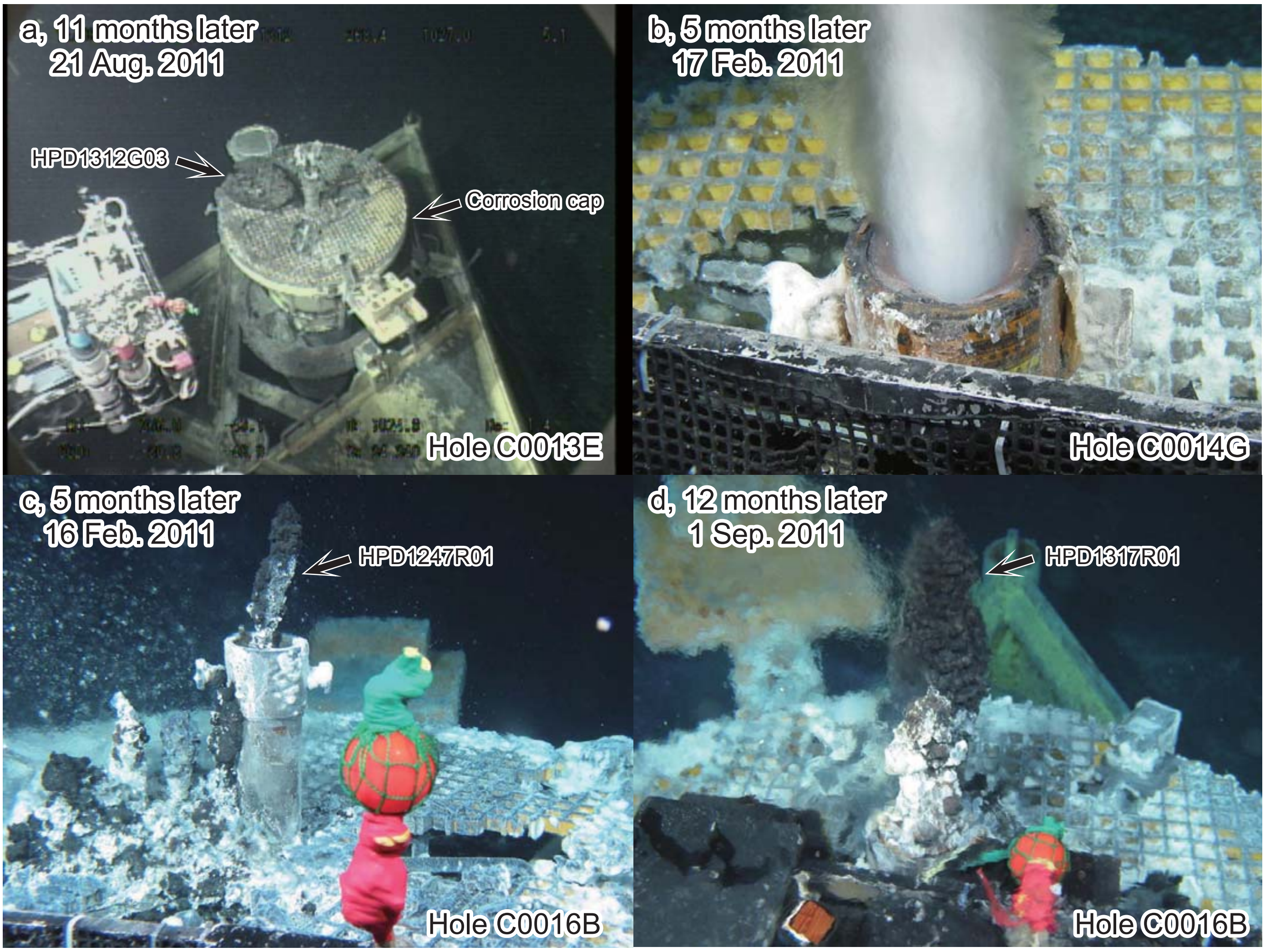

Figure S1 | Photographs of artificial hydrothermal vents. a, Hole C0013E 11 months after drilling. Hydrothermal activity has ceased and a small sulphate-rich chimney (sample HPD1312G03) has grown on the corrosion cap. **b,** Hole C0014G 5 months after drilling. The high flow rate of the hydrothermal fluid has prevented formation of a chimney on the corrosion cap. Hole C0016B **c** 5 months and **d** 12 months after drilling. Hydrothermal activity has continued after drilling and sulphide-rich infant chimney samples (HPD1247R01 and HPD1317R01) were collected. A diameter of the corrosion cap is 49.7 inch (126 cm). Inner and outer diameters of the stainless steel pipe on the corrosion cap is 3.5 and 4.0 inch (8.9 and 10.2 cm), respectively.These camera images were taken by S. Kawagucci, T. Watsuji, J.-I. Ishibashi and K. Takai.

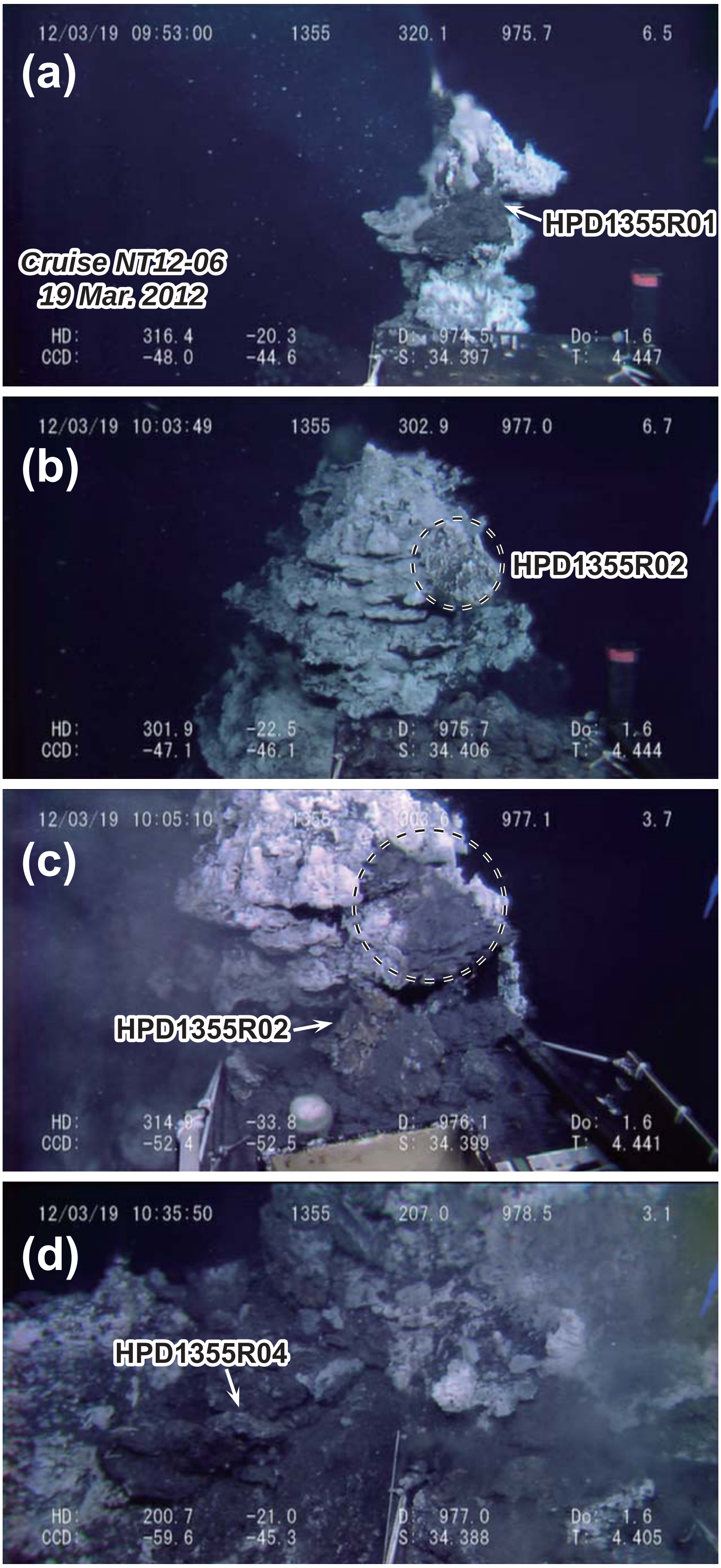

Figure S2 | Photographs of rapidly grown large chimney at Hole C0016A. a, Top part of the active vent chimney. Sample HPD1355R01 was obtained from an anhydrite-rich chimney spire. **b,** Sample HPD1355R02 was a fragment of the 30 cm wide flange. **c,** Cross-sectional image after sampling of HPD1355R02. The inner part of this large chimney was dominated by glossy, black sulphide-rich material. **d,** Sample HPD1355R04 was a chimney fragment taken from the basal part. Because sample HPD1355R03 consisted of fragments that fell into the sample basket during dive HPD1355, precise sampling sites of HPD1355R03 cannot be identified. The width of the sample basket equipped on a ROV (*Hyper Dolphin*) is 76 cm. Photographs were taken by S. Kawagucci, J.-I. Ishibashi and K. Takai.

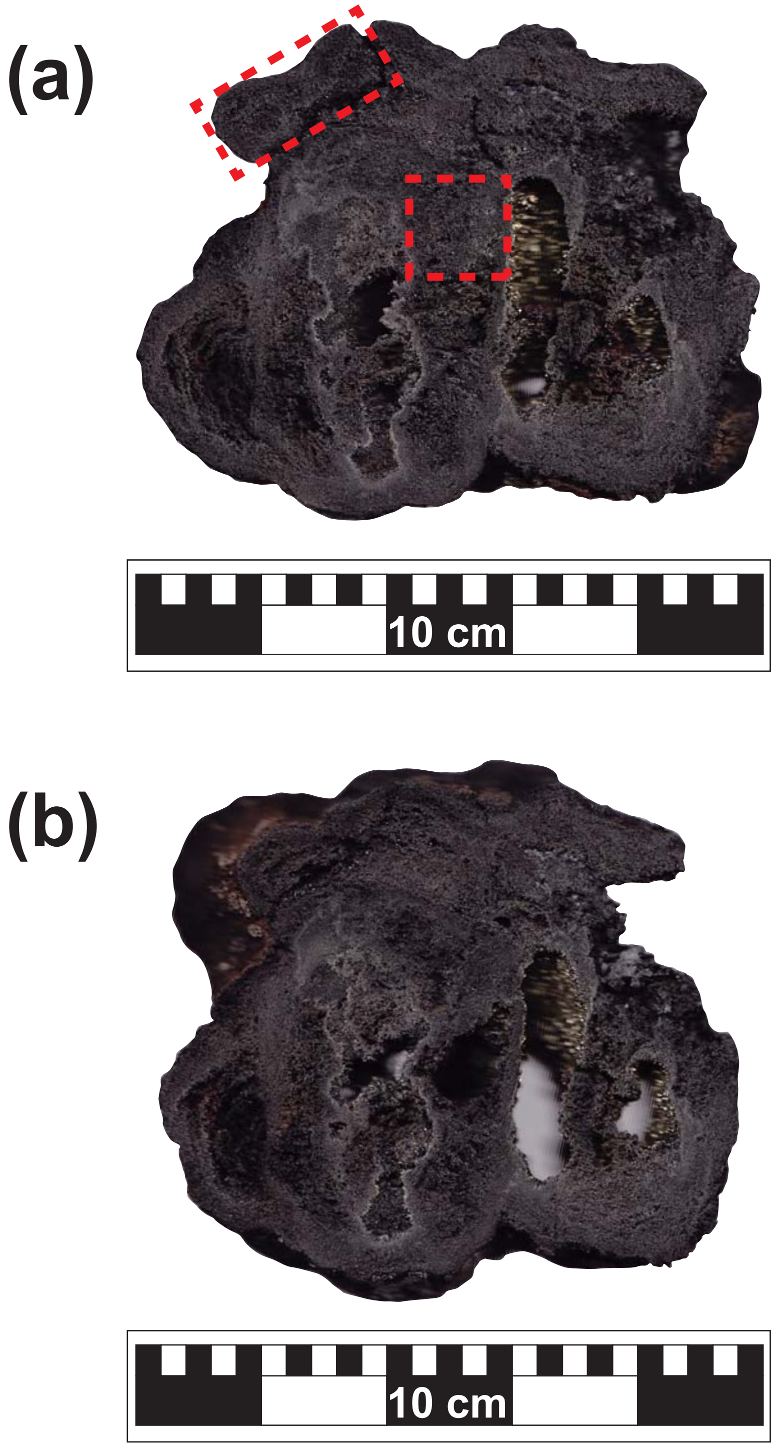

Figure S3 | Scanned images of infant chimney sample HPD1317R01. a, Dashed red outlines denote the inner (right) and outer (left) parts sampled for chemical analysis. **b,** Reverse side of specimen.

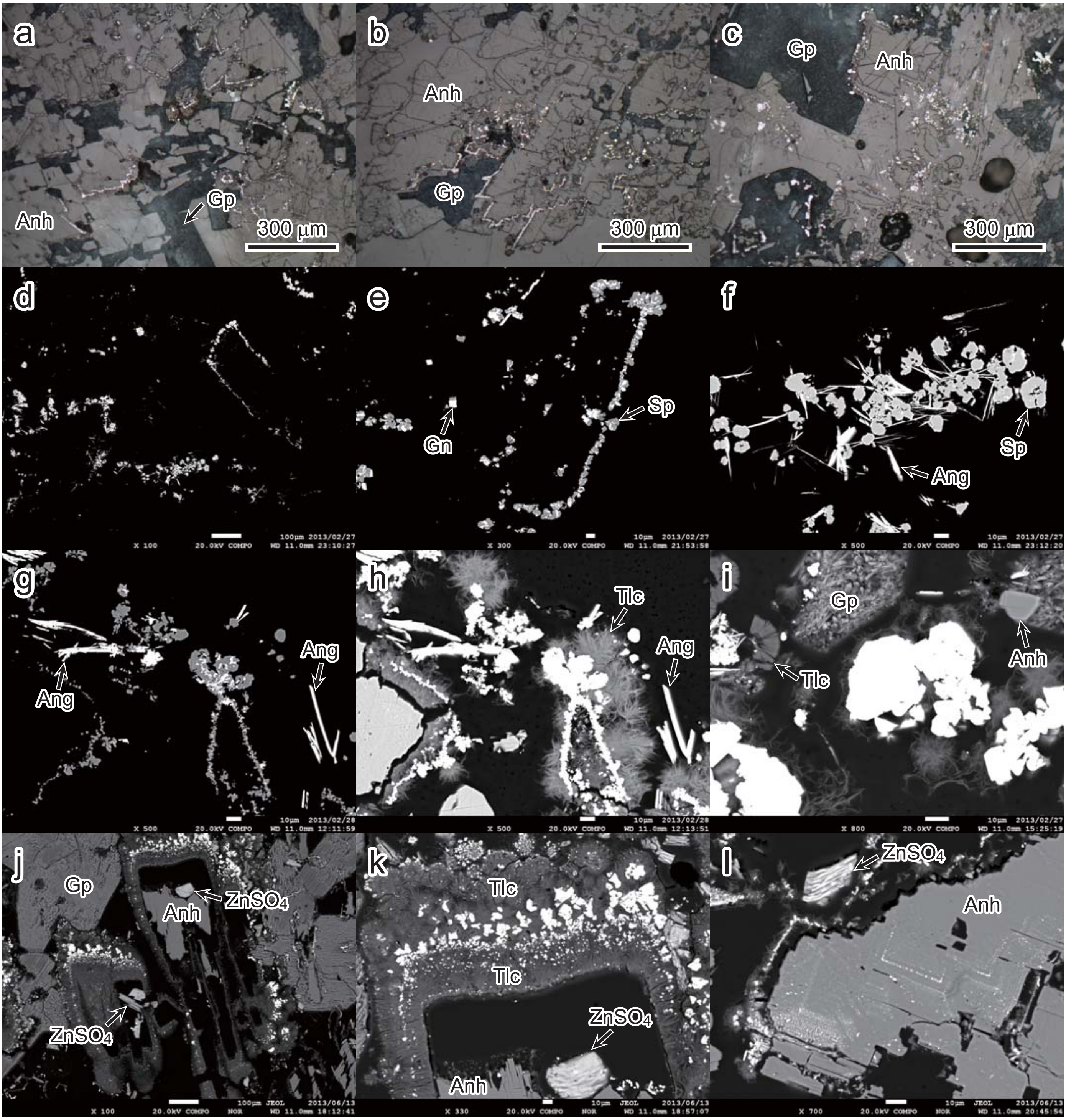

Figure S4 | Reflected-light photomicrographs (a–c) and back-scattered electron images (d–l) of samples from sulphate-rich infant chimneys. a,b,c, Anhydrite with rims replaced by sulphide minerals (sample HPD1312G03). **d,e,** Dendritic sphalerite and galena replacing anhydrite rims (sample HPD1312G03). **f,g,** Cylindrical anglesite precipitated after sphalerite and galena (sample HPD1312G03). **h,i,** (**h**) is the same as **g** with higher brightness. Radiating talc crystals surround dendritic sphalerite and galena. **j,k,** Anhydrite partly dissolved and partly replaced with sulphide minerals. Zn-sulphate minerals occur in dissolution spaces in primary anhydrite (sample HPD1355G01). **k** is an enlargement of the upper middle part of **j**. **l,** Dissolution of anhydrite and replacement of rims by sulphide minerals (sample HPD1355G01). Mineral abbreviations: Ang = anglesite, Anh = anhydrite, Gp = gypsum, Gn = galena, Sp = sphalerite, Tlc = talc.

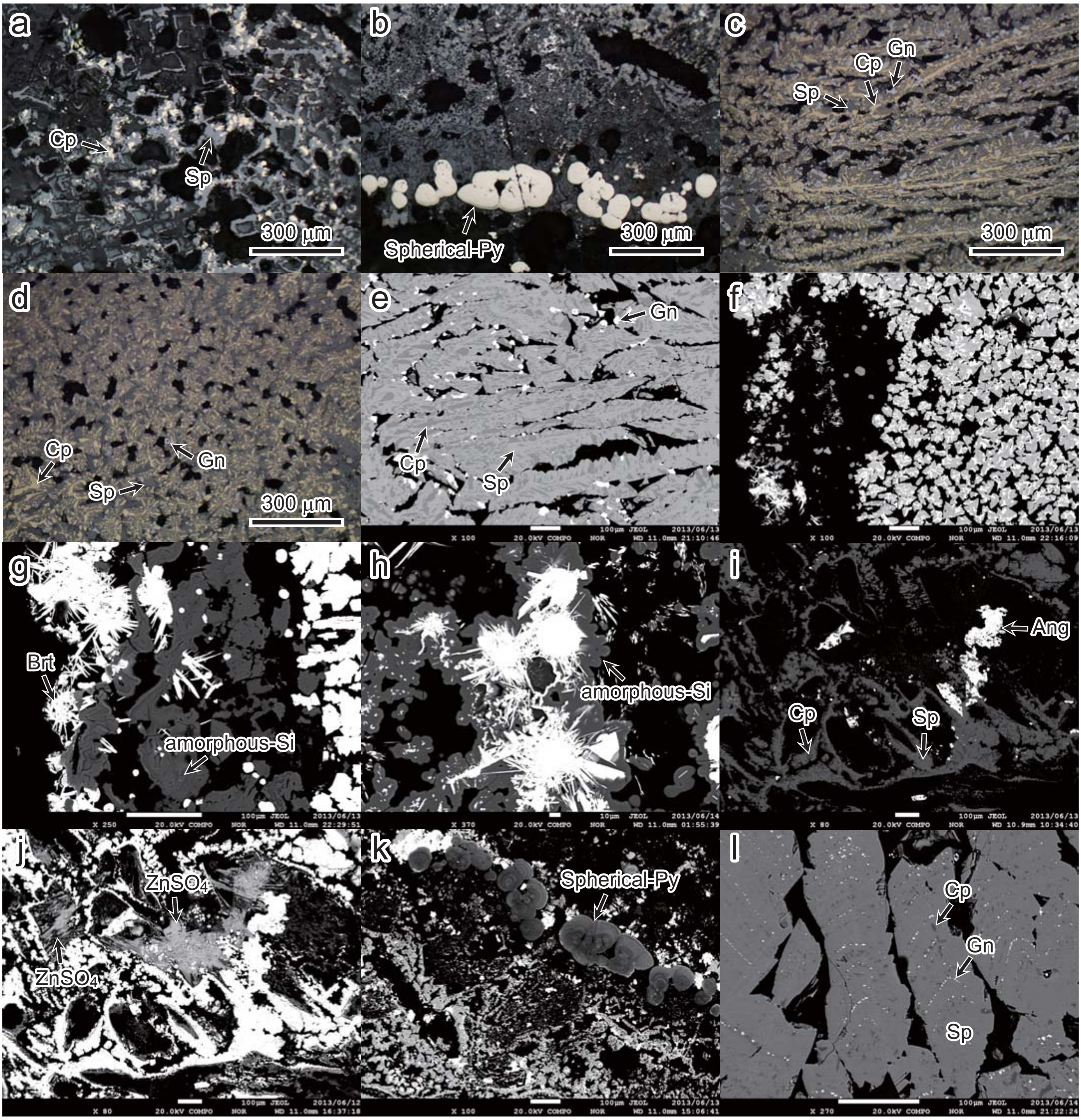

Figure S5 | Reflected-light photomicrographs (a–d) and back-scattered electron images (e–l) of samples from sulphide-rich infant chimneys. a, Encrustation and pseudomorphs of sulphide minerals after sulphate minerals (sample HPD1247R01). **b,** Spherical pyrite crystals in the outermost part of the chimney (sample HPD1247R01). **c,d,e,** Dendritic chalcopyrite, sphalerite and galena (**c,d,** sample HPD1317R01-inner; **e,** sample HPD1449R01). **f,g,** Zoning of constituent minerals from inner (right) to outer (left) side of chimney wall: dendritic chalcopyrite, sphalerite and galena (right), spherical and acicular pyrite (middle), and barite-rich zone (left) (sample HPD1449R01). **g,** Enlargement of the lower-left corner of **f** with higher brightness showing amorphous silica between the dendritic sulphide and barite-rich zones. **h,** Area shown in Fig. 3c at higher brightness. Amorphous silica has precipitated after spherical and acicular pyrite crystals (sample HPD1449R01). **i,j,** Encrustation and pseudomorphs of sulphide minerals on anglesite (sample HPD1247R01). At higher brightness (**j**), Zn-sulphate minerals occupy dissolution voids of Ca-sulphate minerals. **k,** Spherical pyrite crystals with internal zoning in the outermost part of the chimney (sample HPD1247R01). **l,** Intergrowth (eutectic) texture of sphalerite, chalcopyrite and galena (sample HPD1355G04). Mineral abbreviations: Ang = anglesite, Brt = barite, Cp = chalcopyrite, Gn = galena, Py = pyrite, Sp = sphalerite.

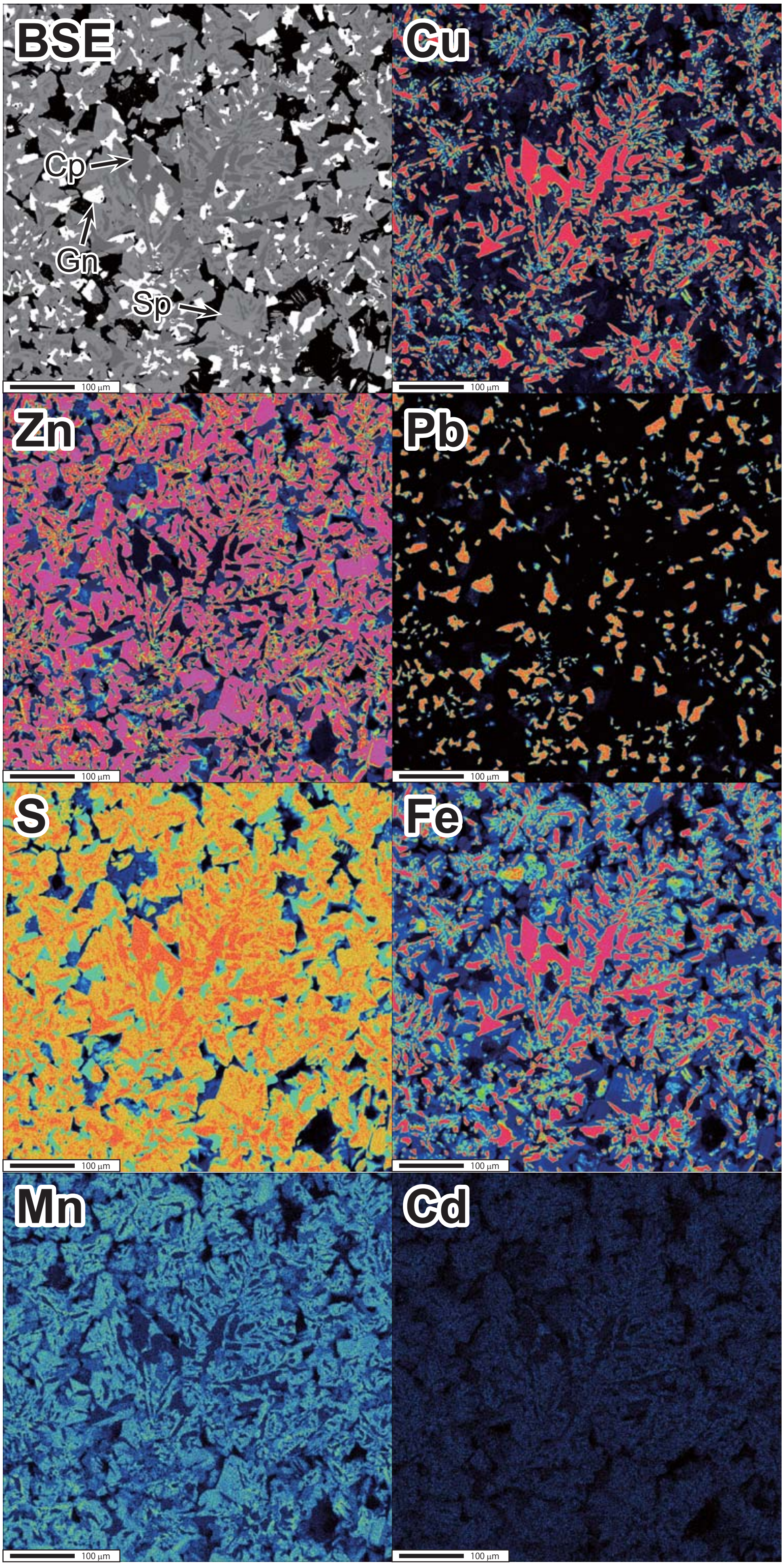

Figure S6 | EPMA maps of dendritic texture in a sulphide-rich infant chimney (sample HPD1317R01-inner). Back-scattered electron image at upper left. Mineral abbreviations: Cp = chalcopyrite, Gn = galena, Sp = sphalerite.

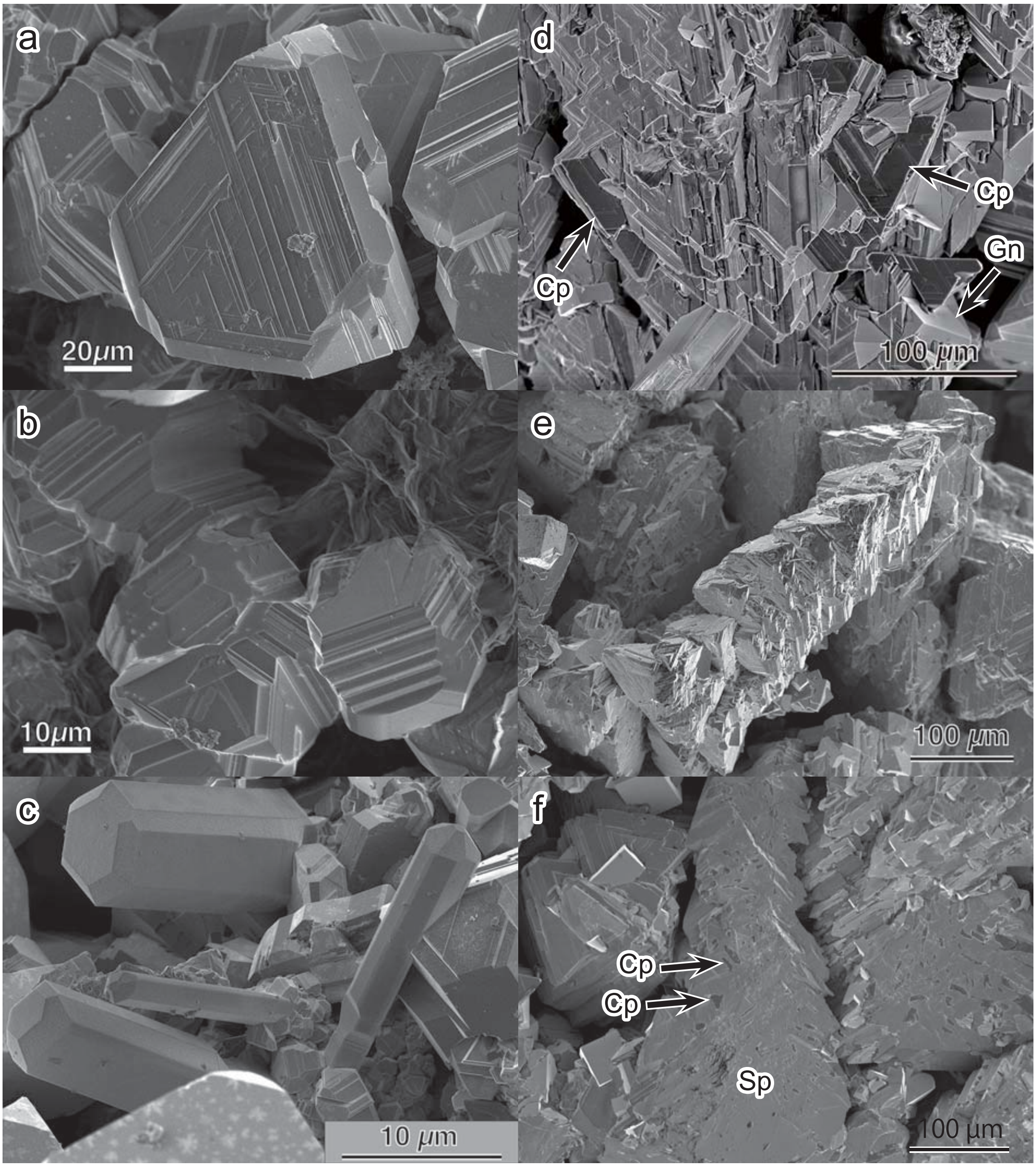

Figure S7 | SEM images of textures and crystal forms in sulphide-rich infant chimneys (sample HPD1449R01). a, Tetrahedral sphalerite crystals with well-developed {111} surfaces and minor $\{\bar{1}\bar{1}\bar{1}\}$ surfaces, rarely associated with wurtzite. **b,** Twinned sphalerite crystals paragenetically associated with wurtzite. **c,** Hexagonal columnal crystals of wurtzite. **d,e,** Dendritic texture of sphalerite, chalcopyrite and galena. **f,** Cross section of dendritic sphalerite crystal showing intergrowth (coprecipitation) texture with chalcopyrite and sphalerite. Mineral abbreviations: Cp = chalcopyrite, Gn = galena, Sp = sphalerite.

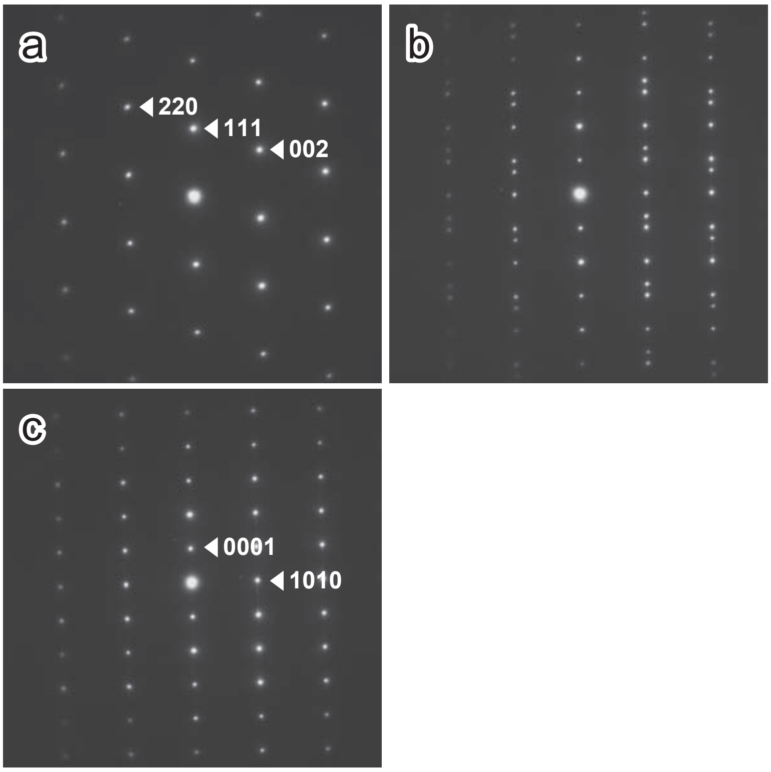

Figure S8 | Selected area electron diffraction patterns. a, Sphalerite. **b,** Twinned sphalerite. **c,** Wurtzite.

Supplementary Tables

Table S1 Compositions of the infant chimneys on the artificial hydrothermal vents determined by ICP-QMS analysis.

Dive No.	HPD1247	HPD1312	HPD1317	HPD1317	HPD1355	HPD1355	HPD1355
Sample No.	R01	G03	R01-outer	R01-inner	G01	G02	G02-B
Hole No.	C0016B	C0013E	C0016B	C0016B	C0016A	C0016A	C0016A
Cruise No.	KY11-02	NT11-16	NT11-16	NT11-16	NT12-06	NT12-06	NT12-06
Drilling depth (mbsf)	45.0	54.0	45.0	45.0	*	*	*
Casing depth (mbsf)	1.0	40.2	1.0	1.0	*	*	*
Time since drilling (months)	5	11	11	11	18	18	18
Na (wt%)	0.34	0.74	0.31	0.05	0.26	0.25	0.40
Mg	0.47	1.57	0.03	0.01	0.44	0.08	0.80
Al	0.02	0.20	0.01	0.001	0.03	0.01	0.11
P	b.d.l.	b.d.l.	b.d.l.	b.d.l.	b.d.l.	b.d.l.	b.d.l.
K	b.d.l.	0.03	0.02	b.d.l.	b.d.l.	b.d.l.	b.d.l.
Ca	8.62	22.7	0.24	0.08	25.2	1.93	23.7
Mn	0.07	0.02	0.39	0.27	0.02	0.27	0.04
Fe	3.84	0.28	8.31	8.50	0.24	7.94	0.49
Cu	1.93	0.19	6.20	7.20	0.15	3.66	0.27
Zn	24.2	1.80	30.8	35.7	1.45	28.9	2.69
As (ppm)	176	3.26	487	116	6.04	518	8.14
Rb	2.88	0.62	1.16	b.d.l.	b.d.l.	0.95	1.46
Sr	309	921	37.9	3.76	962	78.8	1041
Y	0.05	0.39	b.d.l.	b.d.l.	0.16	0.02	0.14
Mo	1.50	0.44	8.84	3.39	0.08	7.21	0.05
Ag	77.0	28.0	455	197	14.5	478	23.5
Cd	669	43.8	850	1065	46.2	712	86.0
Sb	112	11.0	375	135	13.3	740	23.2
Te	6.95	2.77	15.7	13.6	1.18	10.6	1.52
Cs	0.17	b.d.l.	0.15	b.d.l.	b.d.l.	0.13	0.20
Ba[a]	112	255	877	10.6	269	30.2	329
La	0.28	0.89	0.07	0.01	0.78	0.08	0.40
Ce	0.37	1.51	0.04	b.d.l.	1.25	0.03	0.68
W	0.33	0.67	0.15	b.d.l.	1.95	0.07	0.56
Au	0.85	0.10	1.04	0.57	0.12	1.91	0.24
Tl	21.3	0.19	28.4	9.15	0.84	20.5	1.92
Pb (wt%)	0.91	0.71	6.90	7.58	0.17	7.71	0.18
Bi (ppm)	95.7	57.8	147	225	31.9	47.5	44.8
U	0.01	0.11	0.03	b.d.l.	0.03	b.d.l.	0.03

*The core barrel failed during the operation.

b.d.l.: below detection limit.

Abbreviations: B = black part, W = white part, H = hard part, S = soft part.

[a]Ba concentration was possibly underestimated due to precipitation of barite and its insolubility during acid digestion.

Table S1 (continued)

Dive No.	HPD1355	HPD1355	HPD1355	HPD1355	HPD1449	HPD1449	HPD1450
Sample No.	G02-W	G03	G04-H	G04-S	R01	R01-pipe	R01
Hole No.	C0016A	C0016A	C0016A	C0016A	C0016B	C0016B	C0014G
Cruise No.	NT12-06	NT12-06	NT12-06	NT12-06	NT12-27	NT12-27	NT12-27
	*	*	*	*	45.0	45.0	136.7
	*	*	*	*	1.0	1.0	117.6
Time since drilling (months)	18	18	18	18	25	25	25
Na (wt%)	0.47	0.05	0.13	0.58	b.d.l.	0.04	0.31
Mg	0.28	0.10	0.03	4.07	b.d.l.	0.02	0.61
Al	0.01	0.01	0.08	0.54	0.01	0.01	0.11
P	b.d.l.	b.d.l.	b.d.l.	b.d.l.	b.d.l.	b.d.l.	b.d.l.
K	b.d.l.	b.d.l.	b.d.l.	0.09	b.d.l.	0.003	0.05
Ca	25.8	0.05	0.21	13.5	0.07	0.54	27.4
Mn	0.03	0.25	0.27	0.12	0.36	0.34	0.02
Fe	0.35	13.0	8.69	2.66	9.44	10.0	0.22
Cu	0.09	3.97	3.24	1.32	3.93	5.85	0.11
Zn	1.98	34.8	31.2	15.6	30.1	26.9	0.28
As (ppm)	16.2	860	3050	80.5	399	304	6.76
Rb	1.00	b.d.l.	7.65	4.82	0.36	0.13	2.08
Sr	1407	2.05	119	518	1.31	25.8	1279
Y	0.06	0.01	0.02	0.36	b.d.l.	b.d.l.	0.16
Mo	b.d.l.	3.34	32.2	0.51	5.62	8.02	0.70
Ag	16.1	179	524	37.7	273	386	8.75
Cd	44.4	889	701	482	854	867	9.00
Sb	20.6	135	1519	37.9	245	204	14.8
Te	0.37	11.9	12.3	8.16	12.4	13.0	b.d.l.
Cs	b.d.l.	b.d.l.	1.98	0.96	0.08	0.03	0.25
Ba[a]	214	1.11	1920	139	0.97	5.48	323
La	0.13	0.01	0.17	0.43	b.d.l.	b.d.l.	1.07
Ce	0.23	0.02	0.12	0.97	0.01	0.01	1.95
W	0.11	0.11	0.06	0.59	b.d.l.	0.03	0.87
Au	0.16	0.55	5.20	0.49	0.38	0.32	0.04
Tl	2.41	0.81	63.2	5.93	6.31	6.32	0.65
Pb (wt%)	1.21	9.06	9.40	0.52	6.85	6.65	0.08
Bi (ppm)	2.92	164	111	119	61.1	58.1	1.45
U	0.05	b.d.l.	0.38	0.10	b.d.l.	0.004	0.06

*The core barrel failed during the operation.

b.d.l.: below detection limit.

Abbreviations: B = black part, W = white part, H = hard part, S = soft part.

[a]Ba concentration was possibly underestimated due to the precipitation of barite and its insolubility during acid digestion.

Table S2 Composition of unfiltered hydrothermal fluid (sample HPD1356VW) from hole C0016A, determined by ICP-QMS analysis.

Sample HPD1356VW collected at Hole C0016A					
Na (ppm)[a]	7252	As (ppm)	1.00	Ba (ppm)[b]	5.16
Mg[a]	136	Rb[a]	4.57	La	b.d.l.
Al	b.d.l.	Sr[a]	2.76	Ce	b.d.l.
P	0.26	Y	b.d.l.	W	0.047
K[a]	1069	Mo	0.0059	Au	b.d.l.
Ca[a]	1165	Ag	0.016	Tl	0.15
Mn	42.8	Cd	0.010	Pb	1.61
Fe	4.07	Sb	0.071	Bi	0.0020
Cu	0.54	Te	b.d.l.	U	0.00085
Zn	5.07	Cs	1.43		

b.d.l.: below detection limit.

[a]These element concentrations might have been secondarily disturbed by precipitation of salt during evaporation and condensation of the sample solution. Major element concentrations determined by ICP atomic emission spectrometry analysis are given in ref. 10.

[b]Ba concentration was possibly underestimated due to precipitation of barite and its insolubility during acid digestion.
